# Artificial intelligence, fetal echocardiography, and congenital heart disease

**DOI:** 10.1002/pd.5892

**Published:** 2021-02-05

**Authors:** Thomas G. Day, Bernhard Kainz, Jo Hajnal, Reza Razavi, John M. Simpson

**Affiliations:** ^1^ Faculty of Life Sciences and Medicine School of Biomedical Engineering and Imaging Sciences King's College London London UK; ^2^ Department of Congenital Cardiology Evelina London Children's Healthcare Guy's and St Thomas' NHS Foundation Trust London UK; ^3^ Department of Computing Faculty of Engineering Imperial College London London UK

## Abstract

There has been a recent explosion in the use of artificial intelligence (AI), which is now part of our everyday lives. Uptake in medicine has been more limited, although in several fields there have been encouraging results showing excellent performance when AI is used to assist in a well‐defined medical task. Most of this work has been performed using retrospective data, and there have been few clinical trials published using prospective data. This review focuses on the potential uses of AI in the field of fetal cardiology. Ultrasound of the fetal heart is highly specific and sensitive in experienced hands, but despite this there is significant room for improvement in the rates of prenatal diagnosis of congenital heart disease in most countries. AI may be one way of improving this. Other potential applications in fetal cardiology include the provision of more accurate prognoses for individuals, and automatic quantification of various metrics including cardiac function. However, there are also ethical and governance concerns. These will need to be overcome before AI can be widely accepted in mainstream use. It is likely that a familiarity of the uses, and pitfalls, of AI will soon be mandatory for many healthcare professionals working in fetal cardiology.

## Key Points

What's already known about this topic?

- Artificial intelligence (AI) is rapidly becoming part of everyday life, and interest is growing in its use in improving medical outcomes.
- AI may be a useful tool in fetal cardiology to improve rates of antenatal diagnosis of congenital heart disease, as well as other potential benefits.
- Potential pitfalls exist however, and future clinicians will likely need to have a thorough understanding of the risks and benefits of AI.

What does this study add?

- This review article summarizes the fundamentals of AI, the potential uses of AI in fetal cardiology, and what the future may hold.

## 1 ULTRASOUND TO SCREEN FOR CONGENITAL HEART DISEASE: A VITAL TOOL, BUT STILL FAILING

The use of ultrasound to image the fetal heart was first reported in 1964, initially using M‐mode techniques to characterize fetal heart rate and heart size.^1^ The routine use of B‐mode ultrasound to accurately diagnose structural congenital heart disease (CHD) in the fetus began in the 1980s, with groups in the UK and USA publishing case series demonstrating the utility of this technique.^2^, ^3^ Since then, there have been constant incremental technical developments with the introduction of spectral Doppler, color Doppler, and three‐dimensional (3D) imaging techniques, all of which are now in widespread clinical use. Fetal echocardiography in expert hands is a highly sensitive and specific diagnostic test.^4^, ^5^ However, when views of the fetal heart are incorporated as part of anomaly screening programs, both sensitivity and specificity in the detection of CHD are lowered substantially.

Fetal echocardiography is now considered a core component of the routine fetal anomaly scan. Although obstetric practice varies widely, most countries worldwide offer a mid‐trimester fetal anomaly screening ultrasound scan with the aim of detecting serious malformations, and international guidelines recommend that such scans include specific views of the fetal heart.^6^, ^7^ Despite this, antenatal CHD detection rates remain lower than for most other major structural anomalies. International registry‐based data suggest a wide variation in antenatal detection rates, with some countries detecting only 14% of severe CHD cases before birth.^8^ Significant geographical variation within countries has also been demonstrated.^9^, ^10^

This is a problem, because evidence suggests that infants with many forms of serious CHD diagnosed postnatally rather than antenatally are less likely to survive long enough to undergo heart surgery, are less likely to survive after such surgery, and are more likely to have an adverse long‐term neurological outcome.^11^, ^12^, ^13^, ^14^, ^15^ In addition, accurate antenatal diagnosis allows the parents to make an informed decision regarding the continuation of pregnancy, and can also allow therapeutic intervention in selected cases.^16^

There are complex reasons behind the failure of fetal echocardiography to achieve universal antenatal detection of CHD. Recent data have shown that the most frequent reasons for CHD to be overlooked during routine mid‐trimester anomaly scans are poor adaptational skills of the sonographer to acquire and optimize the correct sonographic plane, or failure to recognize an abnormality which is present on the ultrasound image.^17^ Thus, in the majority of cases of missed CHD, either the correct cardiac view was not correctly obtained, or the defect was clearly demonstrated but not recognized by the operator.^17^ Previous work has shown the positive impact of operator experience and programs of staff training to improve recognition of cardiac lesions.^17^, ^18^, ^19^, ^20^, ^21^ However, such programs are labor and time intensive and need to be repeated with staff turnover.

A complementary strategy would be to alter the paradigm, for example by making ultrasound systems “smarter” via the integration of artificial intelligence (AI). Such an approach could have the potential to assist the sonographer in recognizing cardiac abnormalities, whilst remaining unobtrusive, quick, and easy to learn. By combining this approach with continued education and training, it may be possible improve antenatal detection rates of CHD through multiple mechanisms simultaneously.

## 2 CORE CONCEPTS IN AI

AI can be defined broadly as the field of science that aims to use computer programs to learn complex tasks and make predictions based on data.^22^ Although the field of AI research has existed for over 70 years, in the last decade there has been an “AI boom” with extremely rapid progress in multiple fields.^23^ This has been largely driven by three factors: (1) hardware development, with the production of affordable graphical processing units, optimized to perform a huge amount of simultaneous calculations, (2) the growing collection and availability of “big data” (especially data that has been labeled), essential in order to train AI systems, and (3) the application of complex AI methods, including neural networks, which we will discuss below.

**FIGURE 1:**
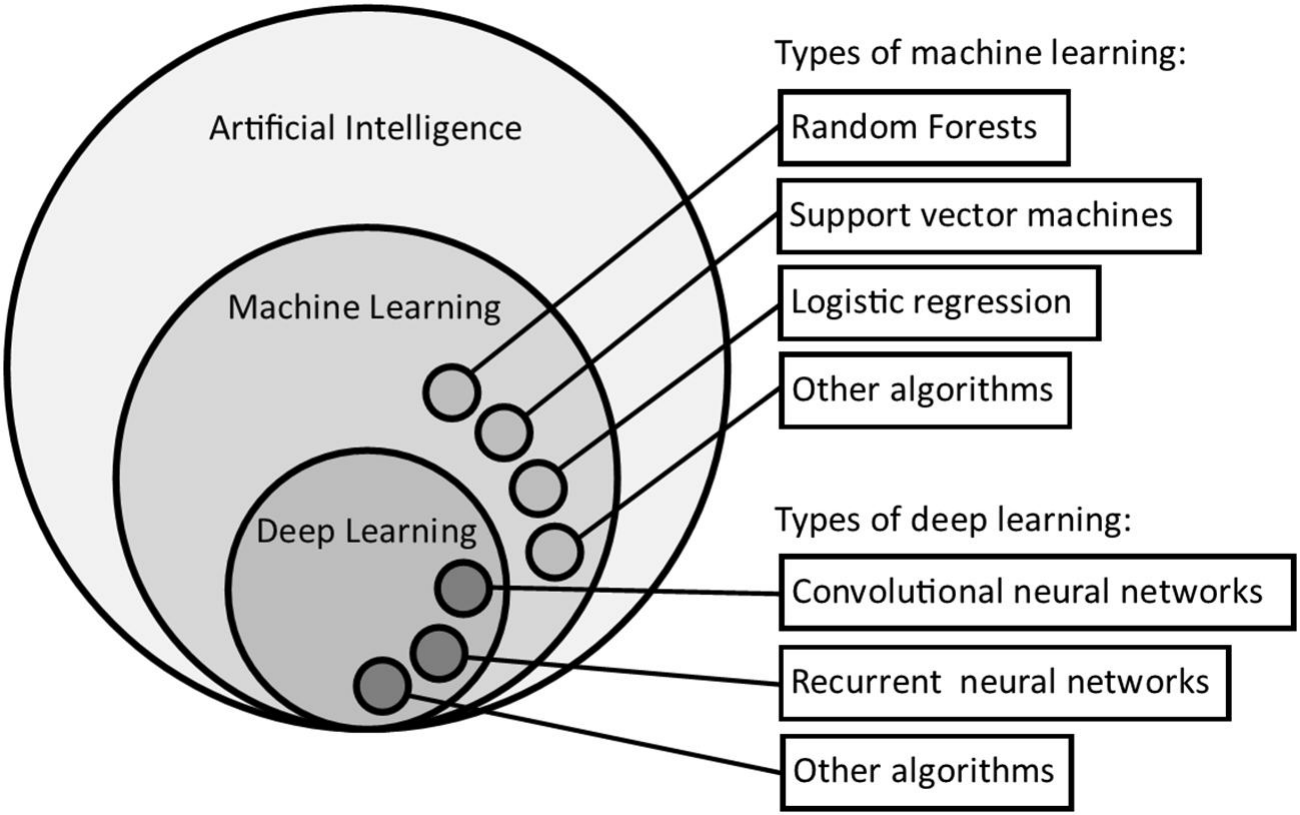
The relationship between artificial intelligence, machine learning, and deep learning, with examples of machine learning and deep learning methods

Machine learning (ML) can be seen as an integral part of AI. ML can be defined as the use of computer programs that automatically improve with experience, so over time will become more successful in their defined task. A more formal way of stating this is to say a machine can be said to learn from experience *E* with respect to a task *T* and performance measure *P*, if its performance of *T*, as measured by *P*, improves with *E*.^24^ Deep learning (DL) is a specific type of ML that uses neural networks (explained below) arranged into many layers (typically more than five, up to hundreds).^25^ Each layer can extract more abstract and high‐level features from the input data, allowing complex interpretation and prediction from the supplied data, for example image classification in the field of computer vision. Figure 1 outlines the relationship between AI, ML, and DL, showing some examples of ML methods such as random forests, support vector machines, and logistic regression, and examples of DL methods such as convolutional neural networks and recurrent neural networks. Many other ML methods have been developed, all which have strengths and weaknesses when applied to a specific problem. A full description of these methods is beyond the scope of this review, but we refer the reader to excellent online learning resources.^26^

ML methods can be broadly categorized into “supervised” and “unsupervised” learning methods. Supervised learning is best used when large amounts of labeled training data are available, and the algorithm uses this data to learn how to make specific predictions when presented with new, unlabeled data. “Unsupervised learning” is a different approach, where the ML algorithm is shown unlabeled data and asked to identify clusters within it, without generating specific predictions. This review is mostly concerned with supervised learning, as this predominates in the medical AI literature, however unsupervised learning can be a powerful tool in the identification of previously unseen patterns within patient data.

Neural networks represent the current state‐of‐the‐art in the field of medical AI, and the development of these models has resulted in super‐human performance in certain medical tasks (although whether this translates to an actual clinical improvement is debatable, as we will discuss later).^27^ There are many varieties of neural networks that have been developed to perform optimally in specific tasks (e.g., convolutional networks for computer vision, recurrent networks for language processing). A full discussion of these network architectures is also outside the scope of this review, but more information can be found at the online learning resources mentioned above.^26^

**FIGURE 2:**
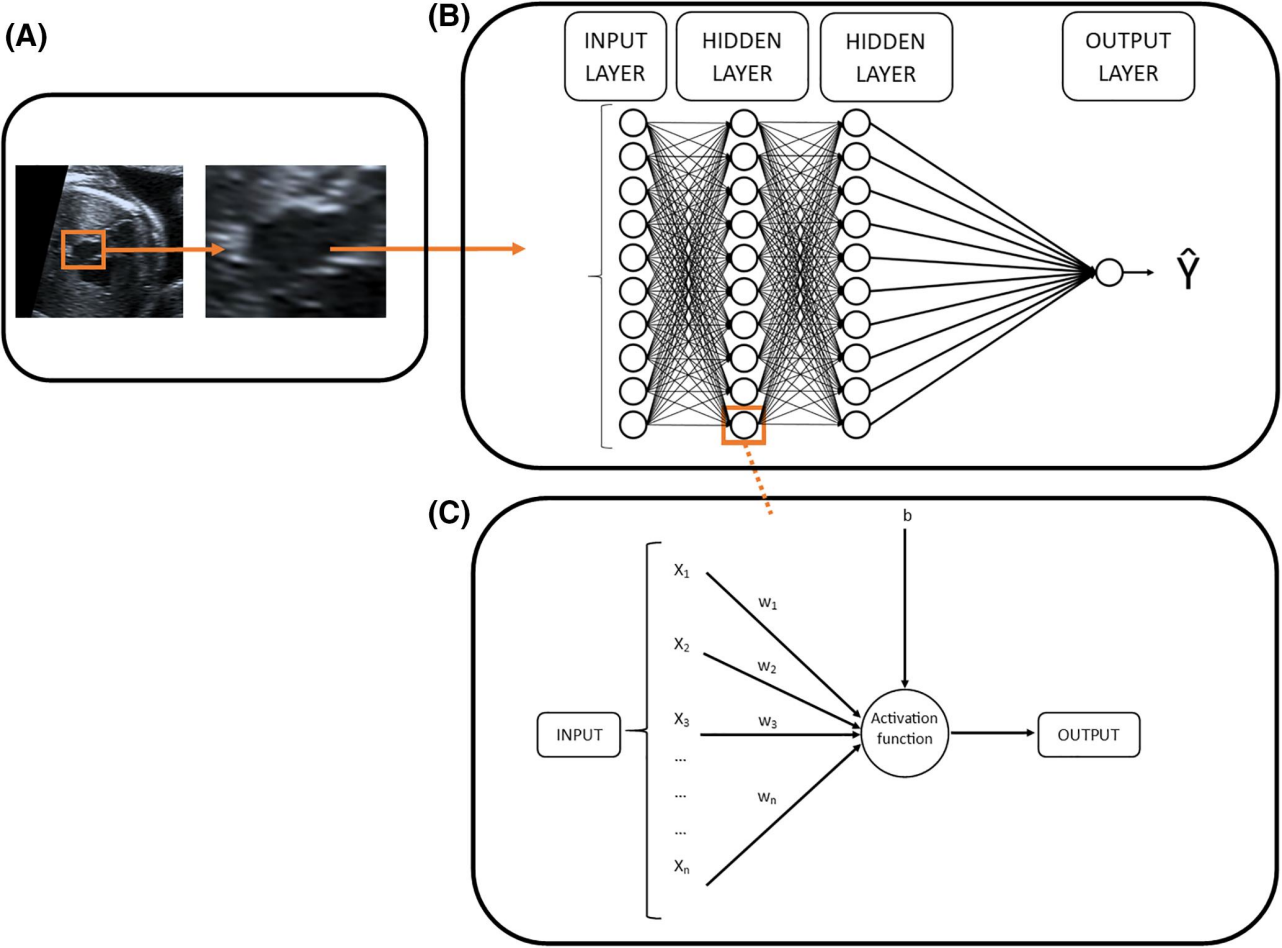
How fetal echocardiogram data (A) might be incorporated into a neural network (B), and how the network is composed of multiple perceptrons (C). *b*, bias; *w*, weight; *X*, input data; *ŷ*, prediction

Neural networks use labeled data (*X*, the raw data; *Y*, the label) to develop a complex model that describes the relationship between *X* and *Y*. The basic building block of the neural network is the perceptron, first described in 1958. A perceptron takes the weighted sum of multiple inputs (*X*), using a vector of weights (*W*), adds a bias (*b*), and passes the resulting sum through an activation function, to give an output.^25^ These weights and biases are known as the *parameters* of the network, and these are what the neural network is learning. Neural networks are composed of multiple perceptrons, arranged into multiple layers, with deeper layers of the network receiving the outputs of the previous layer as an input. Figure 2 demonstrates how imaging data from a fetal echocardiogram might be incorporated into a neural network, and how the neural networks are composed of multiple layers of perceptrons.

Neural networks are trained starting with random values for the parameters. Training involves presenting the network with training data (e.g. image data) and comparing the network predictions to the known ground truth, to calculate an error. Using this error, each parameter can be altered slightly in order to reduce the error. As more labeled training data is passed through, over time the performance of the network will improve in an iterative fashion. The final performance of the network can then be tested by passing previously unseen data (test data) through it and measuring how the predictions compare to the known ground truth values.

## 3 AI IN MODERN MEDICINE

Early attempts to use AI in medicine were disappointing, with rule‐based systems unable to cope with the complexity of medical scenarios, and performed poorly when confronted by large volumes of new data.^22^, ^25^ The development of neural networks has resulted in more success, although real‐world implementations of AI solutions are scarce, with the majority of research papers testing the performance of algorithms using retrospectively acquired labeled data.^28^ In very well‐defined, consistent, and repetitive tasks (such as X‐ray or retinal/skin photograph classification) ML algorithms have been shown to achieve or even exceed human expert‐level performance.^29^, ^30^, ^31^, ^32^, ^33^

Adult cardiology is another area that has shown rapid development in the use of AI, raising hope that fetal cardiology may have similar promise.^35^, ^36^ Both specialties have a large focus on echocardiography, which has the potential to utilize AI to give real‐time feedback to the clinician. Models have been developed that can classify the correct echocardiographic view, and also identify specific pathology within these views.^37^, ^38^

The automatic quantification of cardiac function (in postnatal cardiology) using AI is another area of cardiology that has received much interest, both as a potential to reduce the inter‐ and intraobserver variability seen in current practice, and to reduce the time taken to perform the study.^36^, ^39^ Commercially available solutions that utilize AI are now available.^40^, ^41^ These AI‐based models can sit unobtrusively “on‐cart” within the ultrasound machine, aiding the sonographer by automatically measuring 3D cardiac chamber volumes and ejection fraction. Such methods have been shown to be reproducible, and by reducing or removing human error, provide a means to help standardization across different clinicians or institutions.^39^ There is also the potential to alter and streamline echocardiography workflow, as automated measurements are faster, and allow the sonographer to focus on other aspects of the study.

AI has also shown promise in the context of CHD (the primary focus of fetal cardiology). Using supervised DL, Diller et al.^41^ trained a convolutional neural network to discriminate accurately between patients with discordant ventriculoarterial connections post atrial switch procedure, patients with both discordant atrioventricular and ventriculoarterial connections, and normal controls. Such direct image‐based diagnosis of CHD has exciting potential in fetal cardiology, although with additional challenges. Taking this further, once the diagnosis is known, AI may be able to extract additional information from imaging data and use this to inform prognosis, or perform some other more complex categorization task. As an example of this (although using magnetic resonance data rather than ultrasound), AI has been shown to have utility in the automatic determination of long‐term prognosis in patients with repaired tetralogy of Fallot.^42^ And the potential for AI in this context is not only limited to imaging data. A recent study has shown that by combining clinical data, text from clinical letters (using text mining algorithms) and data from other clinical investigations such as electrocardiography and exercise testing, ML algorithms can be trained to estimate prognosis in a large cohort of adult CHD patients.^43^ As well as predicting outcome, these algorithms can also predict medical interventions, such as the commencement of specific drug therapy. Although not in current clinical use, there is the clear potential in the future for such algorithms in not only predicting, but suggesting, medical treatments and interventions.

## 4 INTELLIGENT IMAGING OF THE FETUS

Table 1 outlines some examples of AI methods and how they have been recently utilized in the field of fetal ultrasound imaging. Given the suboptimal detection of fetal cardiac defects at the screening anomaly ultrasound scan, there may be potential for AI to improve this. AI may also be useful to the fetal cardiac specialist, as although the performance of fetal echocardiography is excellent in expert hands, there are still potential routes to AI improving this performance further. However, fetal cardiac ultrasound imaging is a challenging and complex task. There is a high degree of operator dependency, meaning that different operators may not produce images that are similar in appearance, despite representing the same anatomic area. The operator can vary several parameters such as gain, contrast, resolution, depth, and magnification meaning that the images are not consistent between studies, even of the same patient, and this is compounded by constant fetal movement relative to the ultrasound probe. Imaging artefacts such as acoustic shadowing from bone are common and difficult to avoid, and have the potential to confuse algorithms. Because of the small fetal size, the fetal heart takes up a relatively small proportion of the image (certainly when compared to postnatal echocardiography), meaning that any algorithm will need to learn to ignore a large proportion of the available data. A further difference from postnatal echocardiography is that the orientation and position of the heart in the image is highly variable, creating further complexity in image analysis.

**TABLE 1:** AI methods used in some recent key papers using AI in the field of fetal cardiac ultrasound imaging

AI method	Reference	Training data	Application of AI method
Random forests	Bridge et al.^57^	91 Fetal echocardiogram clips from 12 fetuses	Automatic detection of fetal cardiac position, orientation (axis) and phase
	Le et al.^69^	Echocardiograms from 3910 fetuses (14.1% with CHD)	Differentiation of fetuses with normal hearts from those with a variety of congenital heart defects
Fully connected neural network	Sulas et al.^70^	LV inflow–outflow PW Doppler traces from 5 healthy fetuses	Automatic identification of *E*, *A*, and *V* waves on PW Doppler traces
Recurrent neural networks	Chen et al.^51^	Ultrasound scans of 300 fetuses	Automatic detection of standard planes, including the four‐chamber cardiac view
Convolutional neural networks	Dong et al.^58^	2032 Examples of fetal four‐chamber views and 5000 views of other fetal structures	Automatic detection of the four‐chamber cardiac view, and an automatic assessment of image quality
	Baumgartner et al.^59^	Ultrasound scans of 1003 healthy fetuses	Automatic detection of standard planes, including the four‐chamber, LV outflow, RV outflow, and three‐vessel view cardiac views
	Arnaout et al.^68^	107,832 Images from 1326 ultrasound scans of fetuses (400 with CHD)	Automatic identification of standard cardiac planes, then differentiation between normal hearts and those with a variety of congenital heart defects. Also automatic segmentation of fetal heart structures to allow biometric measurement

Abbreviations: AI, artificial intelligence; CHD, congenital heart disease; LV, left ventricle; PW, pulsed wave; RV, right ventricle.

One approach to improve image acquisition is the use of automated 2D reconstructions of 3D data volumes. In this technique the operator obtains a 3D volume of ultrasound data, including the fetal heart, for example using spatiotemporal image correlation. It is possible to then manually postprocess this dataset, producing 2D images that replicate the standard planes of the fetal echocardiogram, allowing the operator to examine these for evidence of CHD. To some extent this technique removes the need for the operator to be skilled in the subtle and complex movements of the probe that are necessary for the acquisition of standard cardiac planes. However, in effect this just swaps one problem for another, as the operator now needs to become skilled in the difficult task of manually manipulating the 3D dataset to display to 2D images of interest. Techniques have been proposed to automate this process.^45^, ^46^, ^47^, ^48^, ^49^ These could be described as a “human support system” rather than ML, as the system is reliant on a human indicating where specific cardiac landmarks are, then uses predefined rules to reconstruct the required 2D planes. Results have been published demonstrating the potential utility of this technique in fetal cardiac screening.^49^ However, although case reports have shown this technique being of use in detecting isolated cases of CHD, no larger trial demonstrating an improvement in antenatal detection of CHD has yet been published.^50^

The acquisition of standard cardiac image planes is the first step in diagnosing CHD from ultrasound images. Using AI to automatically retrieve these planes from a stream of ultrasound imaging data is a potential route improving detection rates. This may have the potential to reduce the “cognitive load” of the sonographer, allowing them to focus on identification of abnormal anatomy, rather than the pausing and saving of standard panes. The automatically retrieved planes may also be of higher quality than the manually obtained ones, which also may improve diagnostic accuracy. Several groups have investigated this method,^52^, ^53^, ^54^, ^55^, ^56^, ^57^ including some focusing solely on the fetal heart.^58^, ^59^ Our group in collaboration with others has previously published the SonoNet algorithm.^60^, ^61^ This uses a deep convolutional neural network that was trained using labeled routine mid‐trimester ultrasound images from 2694 volunteers. This achieves real‐time classification of standard screening planes from a continuous stream of ultrasound video data. Using this we can automatically save the required images, without the sonographer having to freeze and manually save the image. These images can then be automatically used in a standardized reporting template, further streamlining workflow.

In addition to automatic plane detection, work has been published on automated fetal biometry.^62^, ^63^, ^64^ For similar reasons to above, this could conceivably improve anomaly detection rates by freeing up the sonographer from mundane tasks, who may then be more likely to identify abnormal anatomy. Human‐level performance in measurement accuracy has been demonstrated on retrospectively acquired data. In current practice, we use measurements of cardiac structures indexed to the gestational age of the fetus. If fetal size is automatically determined, then such measurements could be more informatively indexed to the fetal size, and indeed even these cardiac measurements could potentially be automated. In addition to 2D biometric measurements, early work has been published on the automatic segmentation of a 3D volume of ultrasound data.^64^ Although currently this work focuses on identifying the entire fetus rather than specific fetal anatomy, it raises exciting future possibilities of being able to automatically segment the complex 3D cardiac structures, which may be of great use in the identification of fetal cardiac disease.

The quality of the acquired ultrasound images has been shown to relate to the likelihood of clinically significant errors, and this is a particular area of concern in fetal ultrasound scanning as it usually involves lone and siloed working, and it is not standard practice to store the entirety of the ultrasound data stream for later review.^65^ AI has been investigated as a means to automate the quality‐control process. Wu et al.^66^ used deep convolutional neural networks to assess the quality of abdominal views, based on the relative size of the region of interest, and key anatomical structures within it. Focusing on the cardiac four chamber view, Dong et al.^58^ both automatically extracted this standard plane from the ultrasound data stream, and then used convolutional neural networks to assess the image quality (based on appropriate gain and zoom, and appearance of key anatomical landmarks). This automatic quantification of image quality may be a useful adjunct to standard ultrasound scanning performed by humans, but also will likely form an integral part of any more automated system.

Relatively little work has been published examining the use of AI to directly classify fetal hearts into normal or abnormal. Arnaout et al.^67^ ^,^ ^68^ have proposed the use of an ensemble of neural networks to explore this. Using 107,823 ultrasound images from 1326 echocardiograms, they successfully used supervised learning to train a model to identify specific cardiac views. They then used a separate model to differentiate between structurally normal hearts and complex CHD (considered as one group). Good performance was achieved, with an area under the curve (AUC) of 0.99, sensitivity of 95%, and specificity of 96%, which is comparable to expert human clinician performance. Le et al.^69^ have also published similar work in abstract form, with good model performance on detecting CHD based on retrospective data. They trained random forest algorithms (a different form of ML from neural networks) to differentiate between normal hearts and a group of congenital heart defects, using echocardiogram data from 3910 fetuses, achieving an AUC of 0.83, sensitivity of 93%, and specificity of 72%. As an alternative approach, Sulas et al.^70^ have investigated the use of neural networks to automatically interpret pulsed‐wave Doppler traces on the left ventricular inflow and outflow. They postulate that this might be an alternative method of quantifying cardiac time intervals, given the difficulties in obtaining fetal electocardiographic data, although the utility of this technique in identifying fetal disease has not been tested. How to translate all of these findings into community‐level screening using prospectively acquired data, and how well this would work, has not yet been assessed.

Current practitioners of fetal cardiology will know that information given to prospective parents about a particular diagnosis is usually fairly generic, and other than a few well‐described risk factors for poor outcome or need for early intervention, it is difficult to provide a personalized description of the likely life course for each individual fetus. As has been described above for adult CHD patients, AI may be a means of extracting previously unrecognized information from ultrasound imaging data, and perhaps combining this with data from other sources such as other clinical parameters or fetal magnetic resonance imaging. This could provide a prediction not only of diagnosis, but more granular predictions such as life expectancy, need for urgent intervention, and even quality of life long‐term. Such precision medicine could have great potential in the parental counseling process, and inform decisions such as place or mode of delivery of the fetus.

## 5 SHOULD WE BE WORRIED? ETHICS AND CONTROVERSIES

AI has now become a common part of our everyday lives. Take‐up thus far has been more limited in the medical world, partly because of an understandable degree of caution on the part of clinicians when introducing a novel technique into a high‐risk situation. Obstetrics is a particularly litigious branch of medicine, and so it would not be surprising if caution were even higher in this specialty. And indeed, there are good reasons to be wary. If there is an error in an algorithm then the potential for adverse impact is large, either by “missing” cardiac lesions or by increasing false positive diagnoses in a systematic manner, compared to individual human error. Confirming this risk, there have already been cases of commercially available AI tools performing poorly and potentially causing harm to patients, by making incorrect decisions when applied to a real‐world scenario.^71^ This risk is increased by the fact that it is often difficult to identify exactly why neural networks have made a certain prediction, the so‐called “black box” problem.^27^, ^72^ This is a particular challenge in medicine, where it is likely that clinicians and the public will demand a degree of “explainability” before an AI tool is considered acceptable.^28^ It is also possible to inadvertently introduce unwanted bias into an algorithm, for example creating a model that works better in people of certain races, which would clearly be unacceptable for a medical application.^28^, ^73^

The vast majority of medical AI research has been performed using retrospectively acquired data, which has been used both for training and testing the algorithms. Although many authors have published very favorable results outlining excellent model performance, very little work has been done examining how to apply these models to a real‐world setting, on prospectively acquired data. And even fewer studies have demonstrated an actual clinical benefit to patients by using these new tools.^73^ Although a model may show superior performance to an expert clinician in a highly controlled test situation using retrospective data, this by no means translates into superior performance in the chaotic and unpredictable world of clinical medicine. It is very difficult to interpret model performance in these terms unless high‐quality randomized controlled trials are performed in the future. A recent systematic review found only two published randomized controlled trials comparing the performance of AI versus humans in medical imaging tasks, and identified a high risk of bias in the majority of the published literature using retrospective data.^74^

## 6 THE FUTURE

At the present time there is a lack of a robust framework to help design and assess clinical trials of AI, and exactly how these trials should be undertaken is debatable. As we have discussed, there are a variety of ways that AI could be applied to fetal cardiology, ranging from automated measurement of cardiac biometry, through to direct diagnosis of cardiac malformations, with the sonographer using this information to improve their performance. What we are asking of AI will define how models are developed in the future, and how the clinical trials to assess these models are designed.

**FIGURE 3:**
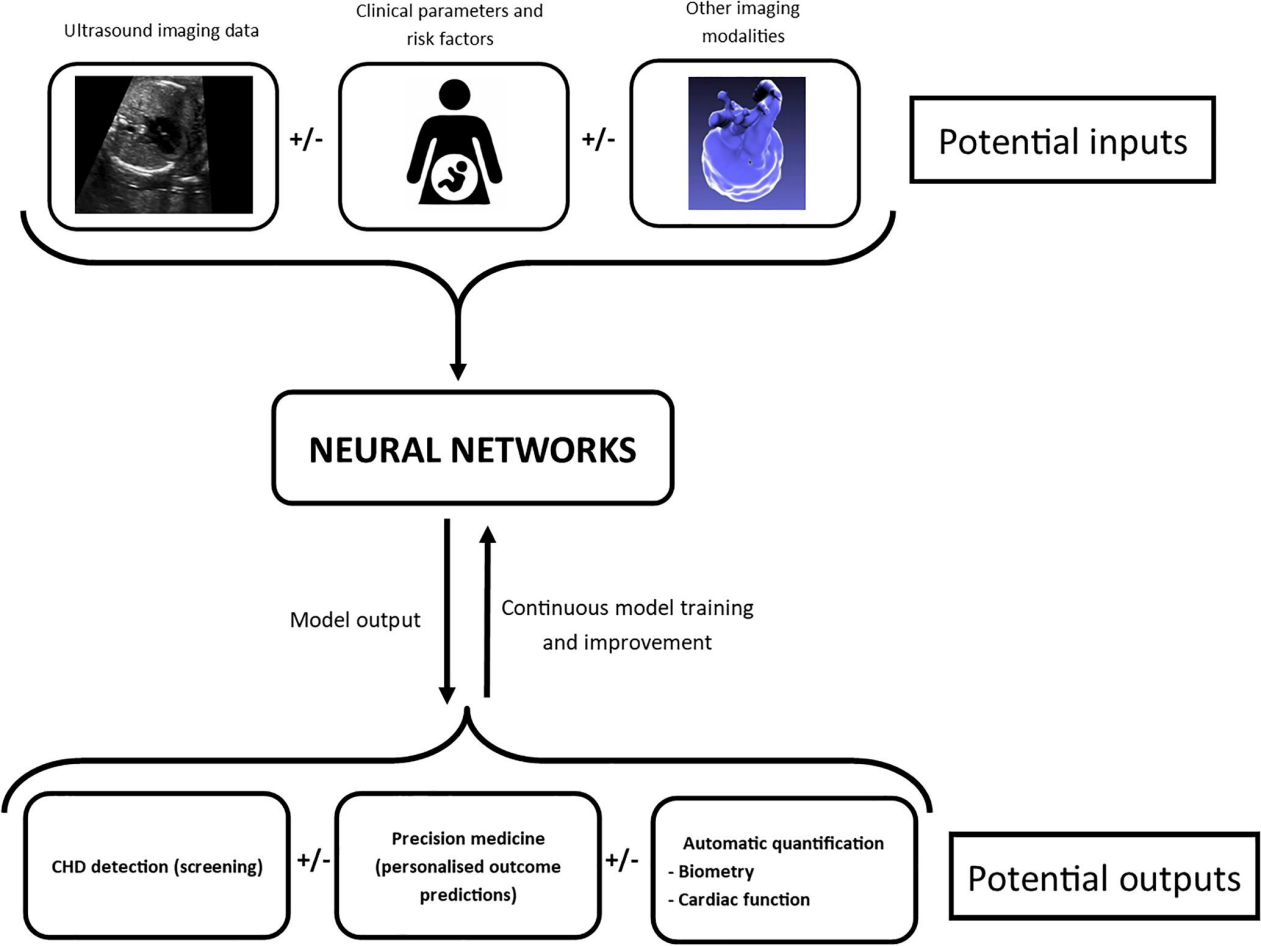
Schematic diagram of how machine learning models could be embedded in fetal cardiology clinic workflow

If we want to train models to detect individual lesions using a supervised approach, then a large amount of training data will be required. Crucially, for supervised learning this will need to be labeled data. For some nonmedical applications of ML it has been possible to use the general public to provide labeled training data (e.g., clicking on pictures that contain specified items), but clearly this would not be possible in fetal cardiology, where a high degree of expertise is needed, creating a scarcity of labeled data that is an impediment to algorithm training. One scenario would be AI algorithms that are completely embedded in the workflow of the fetal cardiology clinic. In this way, labeled data could be continuously fed to the ML algorithms that are continuously improving in their performance. Work has been done specifically exploring this concept in the context of fetal cardiology, and this may be a means by which AI can both inform and learn simultaneously.^75^ However, the regulatory framework in most countries would not currently allow such an approach to be utilized in clinical practice, as approval so far has only been granted for finalized “locked” algorithms, but not for those that are constantly changing. “Catastrophic forgetting,” where an algorithm abruptly deteriorates in its performance on the original task when a new task is learned, is a major barrier to the implementation of such continuous learning.^76^ Nevertheless, the US Food and Drug Administration last year published a discussion paper outlining how such continuously adapting algorithms might be regulated in future clinical use.^77^, ^78^ Figure 3 outlines how neural networks could be embedded in either screening or specialist fetal cardiology workflows, with the potential inputs and outputs of such models.

There may be other solutions to the problem of labeled data scarcity, such as using semi‐supervised approaches (where a model trained on a different task is adapted for a new task by providing a small amount of new training data), or unsupervised methods (where the model sorts the data into similar clusters, then the label is needed only for each cluster). Alternatively, an entirely different approach may be needed where a model is trained to identify “signatures” that are common amongst many types of CHD, and thus identifies fetuses at increased risk. For a truly successful implementation of AI in fetal cardiology, it is likely that novel methods such as these will be required, and this is an ongoing focus of our group's research.

## 7 CONCLUSION

AI shows great promise for future application in fetal cardiology. As a specialty, it faces perhaps greater challenges to realize this promise when compared to other branches of medicine, including the scarcity of labeled data due to the rarity and heterogeneity of CHD, and challenges specific to ultrasound imaging. Nevertheless, once these issues are overcome, along with the development of appropriate regulatory and governance frameworks, it is likely that AI will form at least some part of routine fetal cardiac care in the near future. We envisage a future where AI works in tandem with skilled clinicians to optimize performance. Although the replacement of clinicians with algorithms seems a very long way off, it will likely be within our lifetimes that a familiarity with the uses, and pitfalls, of AI will be mandatory for many healthcare professionals.

## CONFLICT OF INTERESTS

The authors have stated explicitly that there is no conflict of interests in connection with this article.
